# Association of Depression and Anxiety With Diabetes Mellitus Type 2 Concerning Some Sociological Factors

**DOI:** 10.5812/ircmj.12107

**Published:** 2013-08-05

**Authors:** Maryam Palizgir, Maryam Bakhtiari, Alireza Esteghamati

**Affiliations:** 1 Department of Psychology, Islamic Azad University, Najafabad Branch, Isfahan, IR Iran; 2 Department of Clinical Psychology, Faculty of Medicine, Shahid Beheshti University of Medical Sciences, Tehran, IR Iran; 3 Department of Endocrinology, Tehran University of Medical Sciences, Tehran, IR Iran

**Keywords:** Depression, Anxiety, Diabetes Mellitus, Type 2

## Abstract

**Background:**

Diabetes is a metabolic disorder with a high worldwide prevalence. It has been reported that diabetic patients are more prone to depression and anxiety.

**Objectives:**

This study aimed to evaluate the prevalence of depression and anxiety among diabetic patients with regards to some factors such as age, gender, level of education and occupational status.

**Materials and Methods:**

One hundred and eighty four diabetic patients have participated in this study. To assess the severity of depression and anxiety Beck depression inventory and Beck anxiety inventory questionnaire were used respectively. Binary logistic regressions were used to analyze the data.

**Results:**

The results of the present study have shown that 70.7% of the diabetic patients were suffered from depression. Besides, 69.6% of them were diagnosed with anxiety. According to the result, diabetes related depression is affected by sex (OR: 2.767), age (OR: 2.222), level of education (OR: 4.145) and job status (OR: 3.901). It has been also resulted that gender (OR: 2.274), age (OR: 2.706) and Job Status (OR: 2.441) are the effective factors leading to anxiety.

**Conclusions:**

Depression and anxiety have higher prevalence among diabetic patients and some sociological factors such as age, gender, job and education are related to these psychological disorders.

## 1. Background

Diabetes is a worldwide metabolic disorder characterized by chronic hyperglycemia resulting from deficient action of insulin which the main pathophysiological feature is the impaired insulin secretion and increased insulin resistance (1-3). Type II diabetes is caused by both, genetic factors related to impaired insulin resistance and secretion and environmental agents such as obesity, overeating, lack of physical activities, anxiety, and aging (3). The global prevalence of diabetes is constantly increasing. Diabetes is a disease that acquires epidemic form, its frequency has five folded during the last fifteen years which is one of the main threats for human health in the present century (4). Currently, about 285 million people are suffering from diabetes and this amount is likely to increase to 438 million by the year 2030 (more than 70% of the developing countries’ population).Likewise, nervousness and depression have an effect on all populations worldwide and is a global concern (5), it has been reported that the patients with diabetes are approximately twice as likely to suffer from anxiety and depression as the general population (6-8). Previous studies have documented that events of psychological nature are important factors interfering in insulin secretion leading to poor diabetic stability (2). According to evidences, the association between these conditions is bi-directional. It is distinguished that patients with both depression and diabetes, in comparison to diabetic patients alone, have been associated with poor self-care and medical treatment, poorer glycemic control, more diabetes complications. The incidence of depression in persons with diabetes seems to be associated with socio-economic status, family status, obesity, smoking habits, physical activity and sedentary life (9). Furthermore, depression in diabetes patients is linked with a higher risk of morbidity and all-cause mortality (10). It has also observed that both diabetes and anxiety/depression are associated with premature morbidity and mortality (3).

Anxiety is one of the most frequent diseases among all other psychiatric disorders. Knowledge of disease-specific and nonspecific risk factors facilitates the early identification of people at risk, which is important for further treatments (11). Depressive illness in patients with medical disorders is very common. According to the strong relation between depression and other illnesses, there may be useful to use screening tools for depression (12).

## 2. Objectives

The main aim of this study is to asset the prevalence of depression and anxiety among patients with diabetes and finding a correlation between the age, gender, occupation and education and diabetes.

## 3. Materials and Methods

The study has been conducted according to the principles expressed in the Declaration of Helsinki. All participants provided written informed consent of their willingness to be involved. The study was approved by the Medical Ethics Committee of the Shahid Beheshty University of Medical Sciences, Tehran. All diabetic patients referred to the diabetes clinic of the Imam Khomeini Hospital in April to December of 2012 for treatment, aged between 22 and 78 years were invited to participate in our survey (n = 184). In our cross-sectional study the depression, anxiety and HbA1c were evaluated (Biosupply, UK). The severity of depression was determined using Beck Depression Inventory (BDI) Π questionnaire. BDI Π contains 21 questions that assets the severity and existent of depression symptoms and also evaluates the emotional, cognitive and physical symptoms with the score range of 0 - 63. The scores below 4 show the probable denying of the depression and the pretending to be healthy and the very high scores are the indicator of exaggeration in depression, so the patients with these scores were not considered in the present study. Total score of 0 - 13 is considered as No anxiety, 14 - 19 is mild, 20 - 28 is moderate, and 29 - 63 is severe. Also the Beck Anxiety Inventory (BAI) questionnaire was used for designation of presence of anxiety in patients. This test consists of 21 questions and the score is between 0 - 63.

Another questionnaire was prepared and the Patients information such as gender, educational level, duration of diabetes, type of treatment, marital status, occupation, and etc. were reported, these data were evaluated as the associated factors of depression and anxiety. In this questionnaire the educational level were categorized into 2 groups including (diploma degree or less, and academic education), the patients’ ages were divided in 2 groups (22 - 45 years and 46 - 78 years), the occupations were considered as: unemployed and employed and the type of treatment were considered as insulin injection, pill taking or both methods. The glycosylated hemoglobin (HbA1C) levels were measured as the indicator of the blood glucose level in the past few weeks using ion exchange chromatography (HPLC) (Biosystem kit, Spain). Normal levels of glucose produce a normal amount of glycated hemoglobin. As the average amount of plasma glucose increases, the fraction of glycated hemoglobin increases in a predictable way. This serves as a marker for average blood glucose levels over the previous 8 to 12 weeks prior to the measurement. An HbA1c of 6.5% is recommended as the cut point for diagnosing diabetes which reflects the average plasma glucose.

All Statistic analyses were performed using SPSS version 16. Binary logistic regressions were carried out to contrast the data on depressive symptoms, and anxiety and patients with Type II diabetes. In analyses pertaining to depressive symptoms and anxiety, adjusted odds ratios with 95% confidence intervals were obtained separately for patients in different groups (as mentioned previously). We tested whether adjustment for demographic variables (age, education and occupation, etc.) have changed the association of glucose status with depression and anxiety and to determine the effects of some factors on depression and anxiety in diabetic patients. The values were considered significant at P < 0.05.

## 4. Results

**Figure 1.**
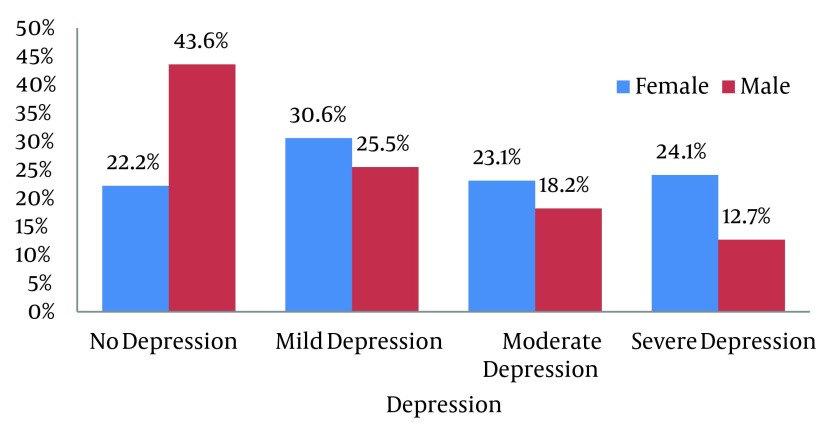
Distribution of Types of Depression According to BDI Test

**Figure 2.**
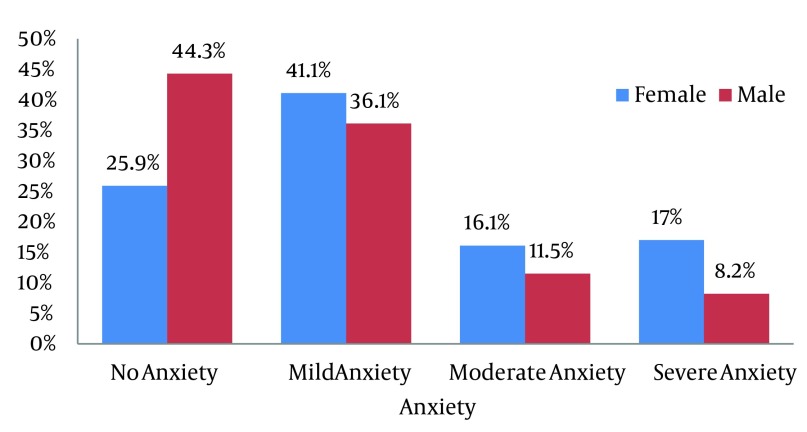
Distribution of Types of Anxiety according to BAI Test

In the present study the average level of HbA1C in patients is 7.49 ± 1.74. According to the results most of the diabetic patients were suffered from depression (70.7%) and anxiety (69.6%). 29.3% had no depression but 28.7%, 21.3 and 20.1% respectively indicate mild, moderate and severe depression. Besides, 30.4% showed no anxiety and 37%, 13.6% and 13% of patients were respectively suffered from mild, moderate and severe anxiety. As shown in Figure 1 and 2, in no depression and no anxiety group men overtook women but in mild to severe depression and anxiety women surpassed men. The demographic characteristics of the patients are presented in Table 1. The effect of some associated factors on depression in diabetic patients is shown in Table 2. 

**Table 1.** The Demographic Characteristics of the Diabetic Patients

Variables	No. (%)
**Age, y**	
22 - 45	69 (37.5)
45 - 78	113 (61.4)
**Gender**	
Male	67 (36.4)
Female	117 (63.6)
**Marital status**	
Single	19 (10.3)
Married	165 (89.7)
**Education**	
Diploma or Less	132 (71.7)
Academic Education	52 (28.3)
**Occupation**	
Housekeeper	95 (51.6)
Governmental job	34 (18.5)
Self employed	25 (13.6)
Retired	30 (16.3)
**Types of treatment**	
Taking pills	137 (74.5)
Injecting insulin	38(20.7)
Both	9 (4.9)
**Duration of diabetes, y**	
≤ 5	72 (39.1)
> 5	112 (60.9)

**Table 2.** Factors Associated With Depression in Diabetic Patients

	Factors Associated With Depression in Diabetic Patients			
	Depression, %	OR	95% CI	P value
**Gender**		2.767	1.385 - 5.528	0.004
Male	56.1			
Female	78			
**Age**		2.222	1.052 - 4.692	0.036
22 - 45	80.3			
46 - 78	64.8			
**Marital status**		0.526	0.172 - 1.605	0.259
Single	57.1			
Married	71.7			
**Education**		4.145	2.003 - 8.578	0.000
Diploma or less	79.2			
Academic education	47.8			
**Occupational status**		3.901	1.909 - 7.969	0.000
Unemployed	83.1			
Employed	56.6			
**Duration of diabetes, y**		0.776	0.384 - 1.568	0.479
< 5	73.8			
≥ 5	68.6			
**Age of onset**		0.988	0.959	1.018
**Types of treatment**				
Taking pill	65.3			
Injecting insulin	83.3			
Both	88.9			

Diabetes related depression is affected by sex (OR: 2.767, 95%CI: 1.385 - 5.528), age (OR: 2.222, 95%CI: 1.052 - 4.692), level of education (OR: 4.145, 95%CI: 2.003 - 8.578) and job status (OR: 3.901, 95%CI: 1.909 - 7.969). The results of the present study have shown that females are more susceptible to depression than males (P = 0.004). In addition the rate of depression was higher in younger patients (P = 0.036) as well as diabetic patients with lower levels of education (P = 0.000). Employment is a preservative factor against depression in diabetic patients (P = 0.000). Table 3 shows the impression of some risk factors for anxiety associated with diabetes. Gender, age and Job Status are the effective factors on anxiety. 

**Table 3.** Factors Associated With Anxiety in Diabetic Patients

	Factors Associated With Anxiety in Diabetic Patients			
	Anxiety, %	OR	95% CI	P value
**Gender**		2.274	1.107 - 4.670	0.025
Male	61.8			
Female	78.6			
**Age, y**		2.706	1.189 - 6.163	0.018
22 - 45	84.2			
46 - 78	66.3			
**Marital status**		0.709	0.188 - 2.675	0.612
Single	78.6			
Married	72.2			
**Education**		0.778	0.358 - 1.689	0.526
Diploma or less	74.1			
Academic education	69			
**Occupational status**		2.441	1.187 - 5.024	0.015
Unemployed	81			
Employed	63.5			
**Duration of diabetes, y**		1.055	0.515 - 2.163	0.884
< 5	72.1			
≥ 5	73.2			
**Age of onset**		0.993	0.962 - 1.026	0.687
**Types of treatment**				
Taking pill	70.1			
Injecting insulin	82.4			
Both	71.4			

Anxiety is higher among females (OR: 2.274, 95%CI: 1.107 - 4.670, P = 0.025). The rate of anxiety is higher in younger patients than the older group (OR: 2.706, 95%CI: 1.189 - 6.163, P = 0.018). Unemployment is another factor increasing anxiety (OR: 2.441, 95% CI: 1.187 - 5.024, P = 0.015).

## 5. Discussion

Following diabetic problems such as type 2 diabetes, some psychological disorders such as anxiety and depression are very common and have a high prevalence among patients. In this study, we have focused on the prevalence of depression and anxiety in patients with diabetes type 2. For this purpose some factors including the age, gender, education, occupational status, the diabetes duration and the type of treatment and the level of HbA1c which is a diabetic indicator were evaluated. Many factors are linked to both Type 2 diabetes mellitus (T2DM) and depression that could be divided to internal factors such as inflammation hormonal status and level of serotonin, and external factors including the occupational status, level of education and gender. It has been observed that inflammation has been associated with the pathogenesis and pathophysiology of both depression and type 2 diabetes mellitus independently, however it has been suggested that depression and T2DM may have common inflammatory mechanisms. An increase in diabetes related inflammatory markers, hyperglycemia and possibly hyperinsulinemia contribute to a net pro-inflammatory state in various tissues. Access of pro-inflammatory mediators to the CNS may then lead to a stimulation of the pathways leading to the progress of depressive symptoms (13). Moreover, T2DM is associated with reduced size of the brain area which would be led to depression such as the hippocampus and amygdala, providing strong evidences for the suggestion that T2DM does provide a true biological risk factor for depression (13).Under stress the body produces hormones, adrenaline being the one we have all heard of and it is often called the fight and flight hormone. These hormones cause the body to release stored glucose and fat for the extra energy that is required to deal with the stress, but they can only be used providing the body has enough insulin. It is this sudden extra production of glucose in people with diabetes that causes the blood sugars to rise. In this paper in addition to other studies, the majority of patients had shown the symptoms of depression and anxiety this results were concluded by using Beck questionnaire (4, 14). The results of this study, in alignment with the reported results by Keita, showed that the prevalence of depression and anxiety were higher among women than men. It is known that depression is 2 folds higher in women than men. This statistic has also been observed among women with diabetes. There are some biological and socioeconomic factors that clarify the reason of higher depression and anxiety of female group such as hormonal changes during pregnancy, postpartum and premenopausal periods, Genetic vulnerability and being dependent to others (15).

The results revealed that younger individuals had higher level of depression and anxiety than the older ones which is closely related to the amount of the patients’ experience in coping with different situations such as their concerns about their treatment procedure and the challenge of diabetes, the influence of disease on physical and psychological functioning and the quality of life resulting from the disease are the factors that increased the ratio of depression and anxiety among younger patients. In a study by Zaho et al. the relationship between the age and depression has been investigated and they have found that younger adults were more likely to have adolescence-onset diabetes and the prevalence of depression and anxiety is 3 time higher than the adolescences without diabetes (16). The educational level is another protective effect against depression. It seems that people with higher levels of education are tending to use health care services than those with lower levels of education (6). Higher Education would help to lower their trend to unhealthy behaviors such as smoking, obesity and fewer tendencies to crime committing (17, 18). The study performed by Bjelland I et al. showed that high level of education is a protective agent against anxiety and depression although in this survey we did not find any relation between anxiety and the level of education.

The occupational status is an impressive element on causing depression and anxiety. Unemployed people are more prone to both anxiety and depression. There are two rational reasons to describe the relation between unemployment, and depression and anxiety, sociological and economical. The unemployed individuals lack sociological functions such as time structure, status and identity, social contacts, participation in collective purposes and regular activity (19). The relationship between unemployment and mental well-being among diabetic patients were assessed in this study and confirmed that the HbA1C level is higher among unemployed patients than employed ones. In this study we have not found any significant correlation between marital status, type of treatment and duration of diseases and anxiety and depression in diabetic patients. In conclusion, diabetic patients are more susceptible to depression and anxiety and some sociological factors such as age, job, education and gender are contributing to these psychological disorders. Therefore, it should be considered in the treatment of the diabetic patients since it can effect on their treatment process as well as the quality of their life.
